# Fast Surface Hydrophilization via Atmospheric Pressure Plasma Polymerization for Biological and Technical Applications

**DOI:** 10.3390/polym11101613

**Published:** 2019-10-04

**Authors:** Hana Dvořáková, Jan Čech, Monika Stupavská, Lubomír Prokeš, Jana Jurmanová, Vilma Buršíková, Jozef Ráheľ, Pavel Sťahel

**Affiliations:** Department of Physical Electronics, Faculty of Science, Masaryk University, Kotlarska 2, 611 37 Brno, Czech Republic; hana.dvorakoval@mail.muni.cz (H.D.); stupavska@mail.muni.cz (M.S.); prokes@chemi.muni.cz (L.P.); janar@physics.muni.cz (J.J.); vilmab@physics.muni.cz (V.B.); rahel@mail.muni.cz (J.R.); pstahel@physics.muni.cz (P.S.)

**Keywords:** polymer surface, polymer modification, deposition, plasma polymer, hydrophilization, superhydrophilic layers, atmospheric pressure plasma, polypropylene, surface free energy

## Abstract

Polymeric surfaces can benefit from functional modifications prior to using them for biological and/or technical applications. Surfaces considered for biocompatibility studies can be modified to gain beneficiary hydrophilic properties. For such modifications, the preparation of highly hydrophilic surfaces by means of plasma polymerization can be a good alternative to classical wet chemistry or plasma activation in simple atomic or molecular gasses. Atmospheric pressure plasma polymerization makes possible rapid, simple, and time-stable hydrophilic surface preparation, regardless of the type and properties of the material whose surface is to be modified. In this work, the surface of polypropylene was coated with a thin nanolayer of plasma-polymer which was prepared from a low-concentration mixture of propane-butane in nitrogen using atmospheric pressure plasma. A deposition time of only 1 second was necessary to achieve satisfactory hydrophilic properties. Highly hydrophilic, stable surfaces were obtained when the deposition time was 10 seconds. The thin layers of the prepared plasma-polymer exhibit highly stable wetting properties, they are smooth, homogeneous, flexible, and have good adhesion to the surface of polypropylene substrates. Moreover, they are constituted from essential elements only (C, H, N, O). This makes the presented modified plasma-polymer surfaces interesting for further studies in biological and/or technical applications.

## 1. Introduction

Since their first adoption, polymers have found broad uses, ranging from technical applications to biological/medical applications [1,2,3]. Bulk polymer materials have convenient chemical and mechanical properties, and can be easily fabricated and deployed. But the effort towards the enlargement of the application basis of polymer materials poses demand on the modification of their surface properties. This ranges from improving surface wettability, the functionalization of surfaces, or deposition of barrier layers, to the fabrication of biocompatible surfaces [4,5,6,7,8,9,10,11,12]. In research focused on biological and/or medical applications of polymers, two principal approaches can be identified. The first one is focused on the synthesis of tailored, “bulk” polymers (see, e.g., [13]). The second is focused on the modification of the surface properties of “standard” polymers (see e.g., [12,14,15,16]).

We can find research on biological applications studying surfaces, polymers, or composites with (highly) hydrophobic as well as hydrophilic properties [17,18,19,20]. And, as stated in [21], discussion is ongoing about, e.g., the dependence of bacterial adhesion upon wettability. The positive influence of the hydrophilization of polymer surfaces on biocompatibility was reported, e.g., in studies on the functionalization of polyestersulfon (PES) membranes for dialysis [15], composites of polymethylmethacrylate and hydroxyapatite in dental implants, [17], and the improvement of cell adhesion on the hydrophilic, plasma-oxidized surface of poly(lactide-co-glycolide) (PLGA) [22]. The positive effect of highly hydrophilic surfaces on the antifouling properties, as well as protein resistance, was also reported [5,23,24,25]. Such antifouling surfaces can be adopted in, e.g., medical [23] or marine applications, such as coatings or sensors [26,27]. In this paper, we would like to introduce a method for the fast and permanent surface hydrophilization of a polymer surface. The method is based on the plasma deposition of plasma-polymeric nanolayers constituted from essential elements only (C, H, N, O).

Common materials like polyethylene (PE), polypropylene (PP), polyvinylchloride (PVC), or polymethylmethacrylate (PMMA) exhibit rather hydrophobic surface properties. The modification of a polymer surface to gain a hydrophilic nature can be done using wet chemistry methods, which could benefit greatly from plasma surface pre-treatment (e.g. immobilizing standard surfactants [28]). The alternative approach for improving wettability can benefit from the lack of wet-chemistry processing. This approach is based on the utilization of gas discharge (plasma) for surface modifications of solid-state surfaces (see, e.g. [29,30]). Direct plasma treatment in atomic gasses (i.e. oxygen, nitrogen, argon, air, etc.) was reported in works [31,32,33], where the introduction of hydrophilic functional groups to the surface was reported. The influence of plasma etching on the surface topography was also reported [29,34,35]. However, there is a significant drawback to the described techniques. The resulting wetting properties depend strongly on treated material properties and treatment (discharge) conditions [36]. A more significant disadvantage is the so-called aging effect, which is the gradual disappearance of improved wetting properties due to ongoing post-treatment surface reactions during sample storage in the ambient gas [37]. Highly-stable hydrophilic or super-hydrophilic surfaces can be prepared using plasma, e.g., by nano-texturizing the surface accompanied by the delivery of new hydrophilic functional groups. However, long processing times [38,39] in low-pressure plasmas are necessary for this method. Further methods of surface activation are plasma-initiated graft polymerization [40,41,42,43,44,45,46] and plasma polymerization [47,48,49,50,51,52,53,54,55,56,57,58,59].

Plasma polymerization is the process of creating a highly-branched polymer by plasma-initiated polymerization of the gas precursor. Typically, plasma polymer is created as a thin layer consisting of short chains with random organization and a high degree of crosslinking [48,60,61]. Low pressure plasma deposition systems were first developed for the preparation of homogeneous and uniform organosilicon, halocarbon, or hydrocarbon plasma polymer thin films. The list of investigated applications includes optical, anti-reflection, abrasion-resistant, and low-surface energy coatings, barrier layers, contact lubricants, dielectric layers, or intermediate adhesive and anticorrosive layers [56,62,63,64,65,66,67]. Low deposition and production rates at low pressure plasmas have motivated the current interest in developing more efficient methods for plasma polymerization using atmospheric pressure (AP) discharges. Depositions at atmospheric pressure allow easy, fast, and continuous processing due to the application of open systems without the need of an expensive vacuum system. The maximum deposition rates of AP systems can be as high as several tens of nm per second [68]. The main drawbacks of AP systems are inadequate uniformity of the deposited films and high precursor consumption.

Even an extremely thin plasma-deposited polymeric layer should be sufficient to provide a significant increase in surface wettability. Therefore, in combination with a suitably-selected AP system, unusually short deposition times can be achieved to significantly improve the aging-free wettability of the coated surface.

The presented fast and cost-effective method for preparing highly hydrophilic surfaces is based on short-time plasma polymerization at atmospheric pressure. We used a low concentration of propane-butane (P-B) diluted in nitrogen as a carrier gas. This gas mixture was known to increase the adhesion of polyester cords to rubber matrices [69]. In addition, this gas mixture is not toxic, nor is it very inexpensive. Deposited thin films are stable, flexible, and they are constituted from the essential elements only (C, H, N, O). Therefore, the presented method can be utilized in biological or technical applications. As potential applications, we can mention the cost-effective production of intermediate layers, biocompatible surfaces that could be utilized in medical applications (e.g. bandages, plasters, device coatings), and marine/water management applications (biofouling control coatings).

The wettability of deposited thin films was characterized by means of the water contact angle (WCA) measurement. The surface free energy (SFE) of thin films was derived from measurements of the WCA and diiodomethane contact angle (DCA). X-ray photoelectron spectroscopy (XPS) and Fourier transform infrared spectroscopy using attenuated total reflection (FTIR-ATR) were used to determine the chemical composition of the deposited layers. Scanning electron microscopy (SEM) imaging was used to characterize the surface morphology.

## 2. Materials and Methods

Commercial polypropylene (PP-H, TUPLEX, Brno, Czech Republic) foil with a thickness of 2 mm, manufactured by extrusion, was selected as a model substrate. Polypropylene (PP) samples with surface areas of 25 × 95 mm^2^ were washed with isopropyl alcohol and dried for 72 hours at room temperature before processing. The water contact angle of the untreated sample was 92° and its surface free energy was 31.9 mJ.m^−2^. A commercial mixture of propane and butane (mass percent composition: 84% propane, 15% butane, and 1% of C2 and C5 hydrocarbons) admixed into nitrogen with a purity of 99.999% was used in this study.

**Figure 1:**
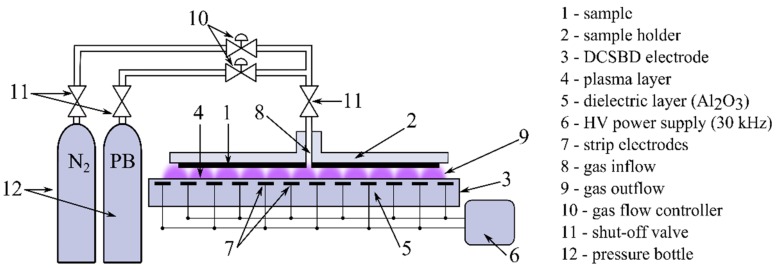
Schematic representation of the experimental set-up.

Figure 1 depicts the experimental configuration of the plasma deposition setup and sample position during the plasma treatment. The flow and composition of the processing gas were controlled by two mass flow meters. The volume concentration of P-B was set at 0.4, 0.8, and 1.2%, and a total gas flow of 3 l.min^−1^ was kept in all cases. The processing gas mixture was injected into the plasma reactor directly in a so-called T-configuration. The so-called Diffuse Coplanar Surface Barrier Discharge (DCSBD) [70] was used to generate plasma for plasma-polymerization. The plasma was generated as a thin layer above the DCSBD surface, using sine-wave high voltage with a frequency of 30 kHz and with a specific power density of 3.75 W.cm^−2^. The PP foil sample was attached to the holder, a moving with a velocity of 15 cm s^−1^ along the DCSBD surface to simulate the conditions of continuous processing. The distance between the sample and the DCSBD electrode was set at 0.1 mm; the treatment times ranged from 0.5 s to 20 s.

The wettability of sample surfaces was determined by measuring the static contact angle using the sessile drop method. The contact angles of two standard liquids (deionized water and diiodomethane) were measured and the surface free energy was evaluated by the Owens, Wendt, Rabel, and Kaelble (OWRK) model [71,72]. Sessile drop of a volume of 1 µl was dropped onto the measured surface and analyzed using See System *E* (Advex Instruments, Brno, Czech Republic). The sessile drop projection was acquired and the contact angles (CAs) were determined. Then, a statistical analysis of the contact angle data, including a surface energy evaluation, was performed using the selected OWRK model. Sets of 10–15 drops of each test liquid/surface combination were used for statistical processing and, prior to CA analysis, the sessile drop was allowed to reach CA equilibrium (ca 30 s).

The chemical composition of the untreated and plasma-treated samples was evaluated via FTIR-ATR and XPS. These analyses were performed on samples treated for 15 seconds. Infrared spectra were obtained with FTIR spectrometer Tensor 27 equipped with a single reflection diamond ATR accessory Platinum ATR (Bruker Optics, Ettlingen, Germany). The XPS measurements were performed on the ESCALAB 250Xi (Thermo Fisher Scientific, Waltham, MA, USA). The X-Ray source was a micro-focused monochromatic Al Kα X-Ray source operating at 200 W (650 microns spot size). The measurements were done under the conditions of 50 eV pass energy and a resolution of 1 eV for a survey and of 20 eV pass energy and a resolution of 0.1 eV for high-resolution spectra. The analysis was carried under an ultrahigh vacuum of 10-9 mbar, at room temperature. To avoid surface charging, an electron neutralizer was used. All binding energies were referenced with respect to C-C/C-H at a binding energy of 284.8 eV.

**Figure 2:**
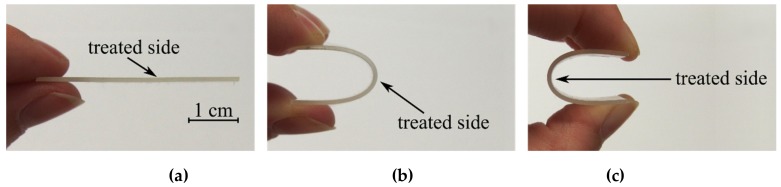
Illustration of the qualitative bending test: pictures (**a**) to (**c**) shows the bending procedure.

The mechanical properties of the layers were studied by means of nanoindentation using a Hysitron TI950 (Bruker Corporation, Billerica, MA, USA) nanoindenter equipped with a Berkovich diamond indenter. Qualitative bending tests were carried out on coated PP foils to study the bending resistance of the samples. The procedure of the qualitative bending test is shown in Figure 2.

The surface morphology before and after the bending test was studied using a Tescan MIRA 3 scanning electron microscope. All samples were coated with a thin conductive carbon layer prior to the observation. Images were taken at magnifications of 50,000 and 100,000 times at a working voltage of 15 kV. Study of the sample surface after the bending test allowed us to study the bending resistance as well as the adhesion of the deposited layers to the PP substrate. To measure the film thickness, scalpel sections were made in the most deformed areas of the layers after bending, which enabled a partial removal of the layer from the substrate surface. The thickness was then measured using the SEM on the side portions of the fragments.

## 3. Results and Discussion

**Figure 3:**
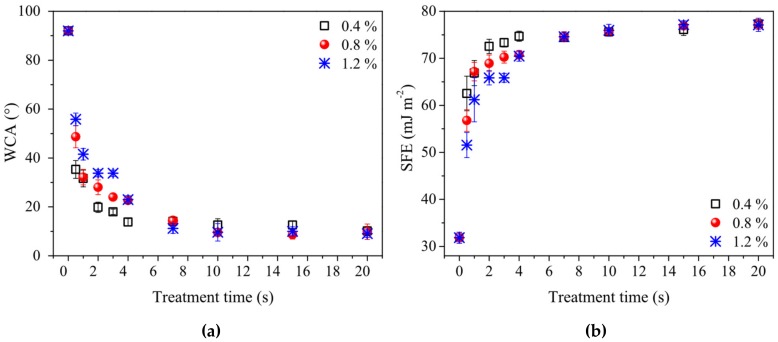
Dependences of surface parameters values on the plasma treatment time: (**a**) water contact angle (WCA); (**b**) surface free energy (SFE). Values for three different compositions of processing gas, i.e. 0.4, 0.8, and 1.2% of P-B in nitrogen, are shown.

The presence of a highly-hydrophilic plasma polymer layer was evident already after short processing times. Figure 3 shows the water contact angle (WCA) and surface free energy (SFE) values as a function of the deposition time for three different processing gas compositions (precursor volume concentration 0.4, 0.8 and 1.2%). The value of WCA dropped sharply already during the first 7 seconds of plasma treatment, then remained constant. Surprisingly, WCA values for samples deposited from the lower P-B concentration in nitrogen converged to the final value more rapidly than in the case of higher precursor deposition mixtures. The values of WCA and SFE of samples deposited for 4 seconds or more were almost independent of P-B concentration. Longer deposition times caused only an increase in the layer thickness. For thicker layers, the scatter of the measured values of WCA and SFE decreased, suggesting that the homogeneity of the deposited layer was increased.

The minimum WCA of 8.7° and the maximum SFE of 77 mJ.m^−2^ were obtained for 20 seconds of plasma treatment in a processing gas of 1.2% precursor concentration. Nevertheless, a WCA value of about 9° was achieved already after 10 seconds of plasma treatment for all tested precursor concentrations. According to [73,74,75], the criteria for superhydrophilic properties are as follows: either a complete wetting of the surface occurs, or the WCA is close to zero on such surfaces. The latter means, in practice, that the WCA is too low for optical recognition, i.e. according to [73,74,75], the WCA < 5–10°. According to these criteria, the prepared surfaces could be classified as superhydrophilic. The SFE components, as well as the diiodomethane contact angles, are given in the supplemental data, Figures S1–S4.

**Figure 4:**
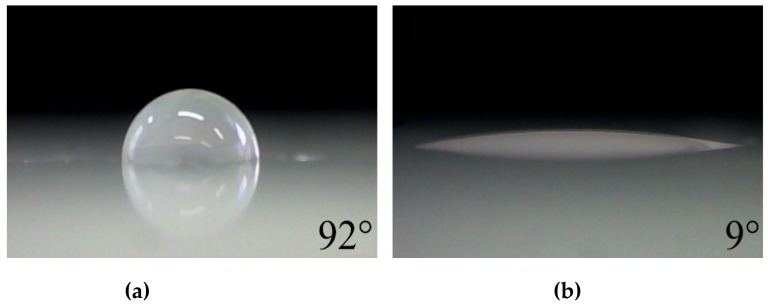
The images of water sessile drop on: (**a**) untreated sample; and (**b**) on the sample after 15 seconds of plasma treatment. Images shown for a processing gas with a volume concentration of P-B of 0.8%**.**

Figure 4 illustrates the visual appearance of the significant decrease of WCA from 92° to 9° after 15 seconds of plasma treatment in nitrogen with a 0.8% admixture of P-B. Without plasma polymerization, the typical values of WCA for plasma-activated polypropylene were within the range of 20° to 70° [76,77,78,79,80,81,82,83,84,85,86]. In general, plasma polymerization makes it possible to achieve much lower values of WCA. For example, the plasma polymerization of acrylic acid (AA), one of the most widely used precursors for hydrophilic modifications, produces surfaces with WCA values of 8° to 43° [45,52,53,54,55]. Using a plasma-initiated graft polymerization of AA on polyethylene terephthalate, a surface with a WCA of 5° with great time stability was prepared; however, the whole treatment procedure took more than five hours [44]. In this context, our results obtained by plasma deposition from the P-B mixture were very encouraging.

**Figure 5:**
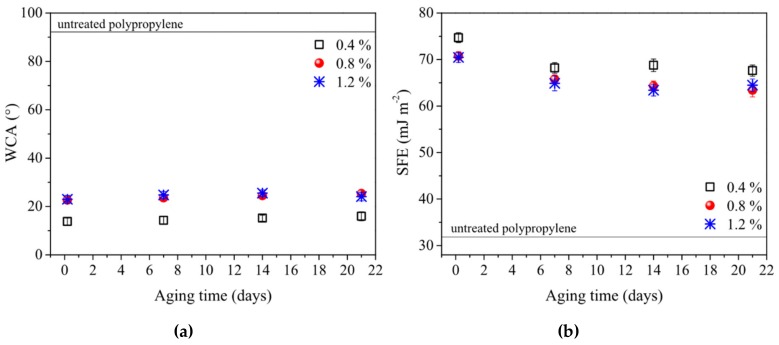
Changes of values (**a**) WCA and (**b**) SFE due to aging effect. Values for three different compositions of processing gas, i.e., 0.4, 0.8, and 1.2% of P-B in nitrogen, are shown. A treatment time of 4 seconds was kept in all cases.

Figure 5 shows the aging characteristics of WCA (a) and SFE (b) for samples treated for technologically-feasible times of 4 seconds. The changes were monitored for 21 days of storage under ambient atmosphere conditions. The WCA increased by 2–3° and the SFE decreased by 4–6 mJ.m^−2^ during the first 7 days of aging; afterwards, their values remained constant. This shows great time stability, especially when compared to the pure plasma activation, in which the wettability improvement decays within a few hours or days [78,79,84,86]. The comparison with other plasma-polymerized or plasma-initiated grafted layers revealed at least equal or slightly better aging characteristics of our P-B layer [40,44,45,59]. The aging of plasma-modified surfaces is a well-known phenomenon described, for example, in [87]. The reversion of the plasma-modified surface properties towards unmodified values was found for both types of modification, i.e., hydrophilization and hydrophobization (see, e.g. [86,88,89]). The aging effect is explained in [87], in which Johansson stated in that thermodynamically driven reorientation, migration of low-molecular-weight additives from the bulk of the plastic to the surface, and airborn contamination of surface should be considered for the aging effect. For the presented plasma polymer thin films, the latter dominates the SFE decrease, explaining the observed decrease from the SFE maximum value being only 7%; see Figure 5. Another mechanism was also reported, i.e., a correlation of surface charges and wetting properties was found [90]. The aging of the surface modification could therefore be influenced also by the plasma-modified electrical properties of the surfaces [91,92,93].

**Figure 6:**
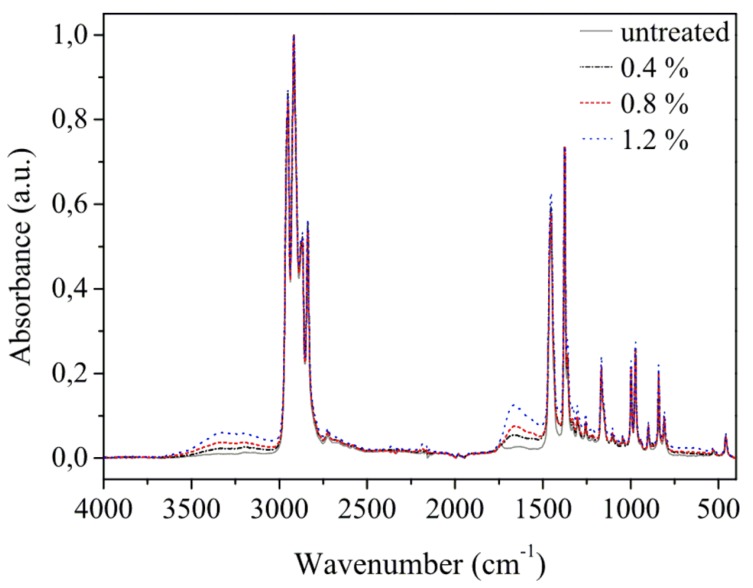
Infrared spectrum of untreated and plasma-treated samples for varied compositions of processing gas, i.e., 0.4, 0.8, and 1.2% of P-B in nitrogen. A treatment time of 15 seconds was kept in all cases.

ATR-FTIR analysis was made on plasma-treated and untreated samples. Spectra were normalized between the minimum and maximum intensity values. The normalized spectra of untreated and plasma-treated samples are compared in Figure 6. In the spectra were identified typical C-H absorption bands related to an isotactic polypropylene substrate [94,95]: 2950, 2917, 2837, 1453, 1376, 1359, 1329, 1304, 1167, 1153, 997, 972, 940, 899, 840, and 808 cm^−1^. In the infrared spectra of the treated samples, two additional wide spectral bands were found. The first one, between 3500 and 3000 cm^−1^, is related to hydroxyl groups of carboxylic acids and a N-H group of amines or amides [96,97]. The second band, between 1730 and 1650 cm^−1^, reflects the presence of carbonyl group of aldehydes, ketones, carboxylic acids and amides, conjugated carbonyls, and imines. Carbonyl groups are often observed in the infrared spectra of polypropylene treated in air plasma [98,99,100] or in methane/oxygen plasma [101]. They can also originate from the post-treatment effect due to atmospheric humidity [57,95,100,102]. The same functional groups were also identified in the XPS spectra.

Due to a high penetration depth (>1 µm) and low layer thickness (maximum around 70 nm), the increase of nitrogen and oxygen group absorption with the increase of precursor concentration is most likely related more to the increase in layer thickness than to real chemical changes in the plasma polymer (the layer thickness is discussed below, see Table 3).

A considerably better detection of surface chemistry is provided by XPS. This method revealed plasma-induced changes in the PP surface composition with a probing depth of 3–10 nm. The estimated minimum film thickness after 15 seconds of plasma treatment was 15 nm; therefore, we assume that the properties of the plasma polymer were measured without the effect of the underlying PP substrate. Atomic compositions for reference and plasma-modified PP surfaces are presented in Table 1. The XPS survey spectrum of untreated PP surface shows that the dominant signals are from C and O, see Figure S5. The initial PP polymer surface contains a low quantity of oxygen (7 at %).

The plasma modification resulted in the reduction of the C1s peak to approx. 70%. As the concentration of the precursor increased, the intensity of the C peak decreased, and the nitrogen content increased, indicating the growth of the nitrogen containing surface layer. The XPS data of all PP modified surfaces show nitrogen incorporation into the deposited carbon layer. The most pronounced difference in the surface composition of the untreated PP sample and the coated PP surface is the appearance of a significant N1s peak, see Figure S5.

**Figure 7:**
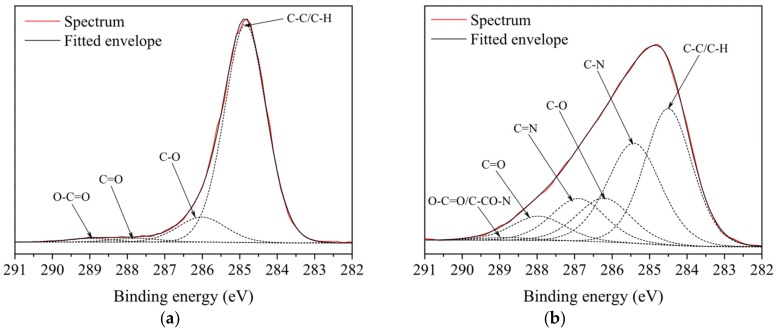
C1s high resolution scan of: (**a**) untreated sample; and (**b**) sample treated for 10 seconds in processing gas with volume concentration of precursor of 0.8%.

The high resolution C1s peak was fitted with 6 principal components: C–C/C–H (binding energy at 284.8 eV), C–N (285.7 eV), C–O (286.3 eV), C=N (287.1 eV), C=O (287.8 eV), and O–C=O/C–CO–N (289.1 eV), which is consistent with the FTIR-ATR measurements and previous reports [103,104]. The results of the fits are presented in Table 2 and Figure 7. As shown, the most significant changes after the plasma treatment for PP are the significant increase of the C–N component and the decrease of the C–C/C–H component. The plasma modification resulted in a massive reduction of the hydrocarbon peak to 35% for 1.2% of P-B. Components which correspond to hydrophilic functional groups containing nitrogen (C–N, C=N and C=O–N) were introduced on the PP surface by means of aforementioned plasma modification.

The most significant change, i.e., 36%, was seen in the increase in the C–N component (1.2% P-B). In the case of plasma-treated PP samples, the peak areas of C–O, C=O, and O–C=O all increased by a factor 1.5–2 compared to the untreated PP. The relatively low oxygen content is not surprising, since the processing gas did not contain oxygen. Oxygen can be incorporated into the structure of the plasma polymer due to post-treatment reactions with ambient atmosphere or air humidity [68,70], or it may be due to a contamination which occurred while using the open plasma reactor. Table 2 shows that the proportion of C–C/C–H bonds decreased with increasing concentrations of precursor in the processing gas, while the proportion of C–N bonds increased. These results correspond to the WCA (Figure 3a); however, it must be noted that the differences in the value of WCA were very small, i.e., within the measurement errors.

As can be seen from the data in Table 1, Table 2 and Table 3, the composition and surface chemistry did not change significantly in the two weeks after plasma treatment, which is in a good agreement with the time stability of the wetting properties (see Figure 4).

**Figure 8:**
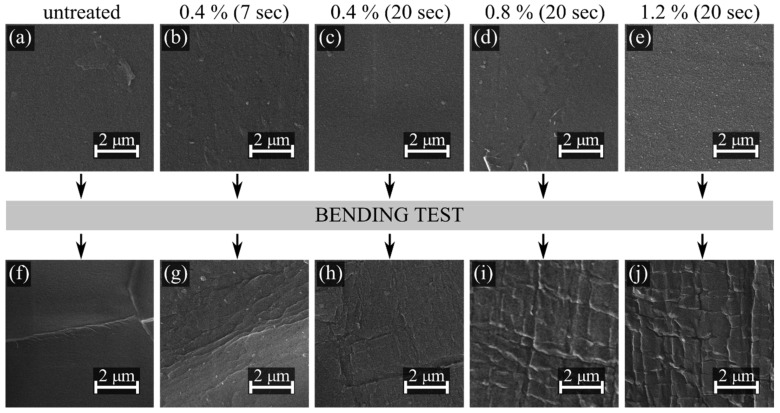
Surface topography of untreated and plasma-treated surface before the bending test; top row (**a**) to (**e**); and after the bending test: bottom row (**f**) to (**j**). The treatment time was 7, resp. 20 seconds and the composition of processing gas was 0.4, 0.8, resp. 1.2% of P-B in nitrogen.

Figure 8 shows SEM images representing the changes in the surface morphology after plasma treatment and after the bending test. Fine structures on the surface are an artefact from the carbon coating. The surface of plasma-treated samples was smooth, homogenous, and without pinholes or protrusions for all treatment times and for all concentrations of precursor. In fact, there were no differences between the surface topography of the untreated and plasma-treated samples (Figure 8a–e). On the other hand, significant differences were observed between samples subjected to the bending test (Figure 8f–j). The untreated sample showed only a local accumulation of material on the surface and the creation of wrinkles. In contrast, the layers deposited for 20 seconds were intensively wrinkled (and partially cracked) after the severe bending test (see Figure 2). The degree of wrinkling and cracking increased with the concentration of precursor, as shown in Figure 8h–j, mainly because of the increase in film thickness. No cracks were observed on the sample deposited for 7 seconds at 0.4% P-B concentration; however, significant wrinkles were created on the surface after the bending test. With an increase of P-B concentration, the degree of wrinkling and cracking increased, as the film thickness became comparable to 20 s deposition conditions; see Figure S6 and deposition rates in Table 3.

**Figure 9:**
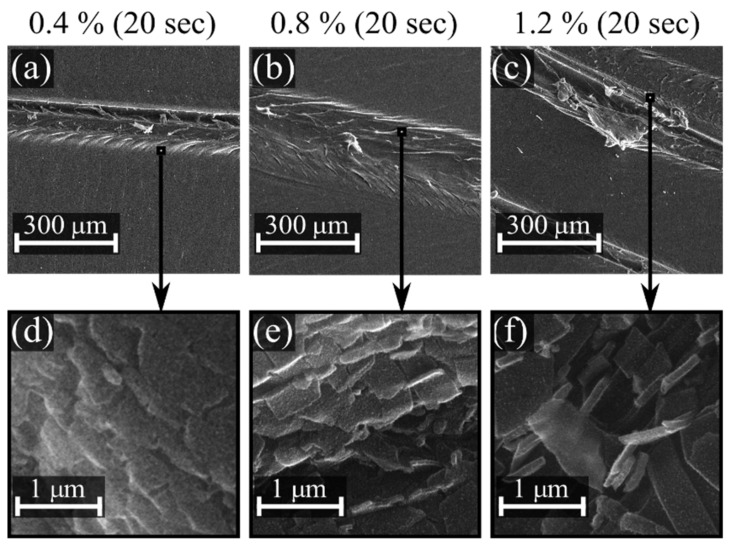
Surface topography of the scratched area for varied composition of processing gas, i.e., 0.4, 0.8, resp. 1.2% of P-B in nitrogen. A treatment time of 20 seconds was kept in all cases. The bottom row represents a magnified view of the surface from the area indicated on the corresponding top row images.

The layers were not delaminated from the PP substrate except for the regions scratched with the scalpel (Figure 9a–c). The fragments separated by the scalpel (Figure 9d–f) were used for the measurement of layer thickness and for the linear estimation of the average deposition rate. The measured thickness of coatings, growth rate, and the concentration of precursor are summarized in Table 3.

The prepared thin plasma polymer layer is smooth and homogeneous, as shown in Figure 8. The layer thickness increased with the processing time and precursor concentration. The peel test showed that the layer exhibited excellent adhesion to the substrate and, in the case of the lower thickness, is relatively flexible and resistant to delamination and cracking, even in the case of severe mechanical stress.

## 4. Conclusions

A new hydrophilization technique based on a plasma deposition of a thin film from mixtures of propane-butane with nitrogen at atmospheric pressure was proposed and successfully tested. Unlike simple plasma treatment, the observed high surface free energy values are due to the properties of the deposited plasma-polymer nanolayer. Therefore, the wettability improvement does not depend on the substrate material, and the aging of the surface modification is highly reduced. The deposited polymer-like layers have proven to be very homogeneous, showing uniform thickness. The measured surface free energy of the coatings was in the range of 60–77 mJ.m^−2^, depending on the coating process conditions. FTIR spectra showed the organic film structure and the presence of C–N and C–C/C–H bonds, as well as C–O, C=O, and O–C=O bonds. The results of XPS analysis were in good accordance with the FTIR observations, confirming also the presence of C–O, C=O, and O–C=O bonds. The basic character of the film surface determined by the surface energy measurement agrees well with the nitrogen-containing hydrophilic groups detected in the surface structure of the films. The films exhibited homogeneous coverage of polymeric substrate and highly hydrophilic properties. With WCA < 10°, the films could be classified as superhydrophilic [73,74,75]. Moreover, the hydrophilized surfaces were composed of essential elements only (C, N, O, H), and as such, their properties could be of interest for utilization in biological, as well as technical, applications.

## Figures and Tables

**Table 1:** Atomic concentrations (at %) and element ratios of untreated and plasma-modified surfaces for three different compositions of processing gas, i.e., 0.4, 0.8, and 1.2% of P-B in nitrogen. XPS spectra were measured immediately after plasma treatment and after 14 days of aging in ambient air. A treatment time of 15 seconds was kept in all cases. The percentages shown are the mean values of three determinations.

Propane-Butane Concentration (%)	Aging Time (Days)	Atomic Composition (at %)			Ratio		
		C	O	N	O/C	N/C	(O+N)/C
untreated PP	-	93 ± 2	7 ± 2	0	0.08	0	0.08
0.4	-	68 ± 2	12 ± 1	20 ± 1	0.18	0.29	0.47
0.8	-	68 ± 1	9 ± 2	23 ± 2	0.13	0.34	0.47
1.8	-	75 ± 1	8 ± 1	17 ± 2	0.11	0.23	0.33
0.4	14	68 ± 1	13 ± 1	19 ± 1	0.19	0.28	0.46
0.8	14	69 ± 1	11 ± 1	20 ± 1	0.16	0.29	0.45
1.2	14	76 ± 1	10 ± 2	14 ± 1	0.13	0.18	0.32

**Table 2:** Percentage (%) of peak areas (functional groups) in C1s peaks of untreated and plasma-modified surfaces for three different compositions of processing gas, i.e., 0.4, 0.8, and 1.2% of P-B in nitrogen. XPS spectra were measured immediately after plasma treatment and after 14 days of aging in ambient air. A treatment time of 15 seconds was kept in all cases.

Propane-Butane Concentration (%)	Aging Time (Days)	Functional Groups (%)					
		C−C/C−H	C−O	C=O	COO/C−CO−N	C−N	C=N
untreated PP	-	87	9	2	2	0	0
0.4	-	45	9	10	3	21	12
0.8	-	36	12	8	1	29	14
1.2	-	35	12	5	2	36	10
0.4	14	48	8	10	5	17	12
0.8	14	36	12	9	2	28	13
1.2	14	36	12	6	3	32	10

**Table 3:** Dependence of thickness of coatings of samples deposited for 20 s and calculated deposition rate on the volume concentration of P-B in processing gas.

Propane-Butane Concentration (%)	Thickness (nm)	Deposition Rate (nm.s^−1^)
0.4	23 ± 4	1.2 ± 0.2
0.8	44 ± 2	2.2 ± 0.1
1.2	94 ± 7	4.7 ± 0.4

## Supplementary Materials

The supplementary material is available online at https://www.mdpi.com/2073-4360/11/10/1613/s1, Figure S1: Dependences of diiodomethane contact angle (DCA) values on the plasma treatment time. Values given for three different compositions of processing gas, i.e. 0.4, 0.8 and 1.2% of P-B in nitrogen are shown, Figure S2: Dependences of surface free energy (SFE) values and corresponding LW, resp. AB components on the plasma treatment time for a gas mixture of 0.4% of P-B in nitrogen, Figure S3: Dependences of surface free energy (SFE) values and corresponding LW, resp. AB components on the plasma treatment time for a gas mixture of 0.8% of P-B in nitrogen, Figure S4: Dependences of surface free energy (SFE) values and corresponding LW, resp. AB components on the plasma treatment time for a gas mixture of 1.2% of P-B in nitrogen, Figure S5: XPS survey spectra of the surface of ‘as-received’ and after thin film deposition for 10 seconds in a gas mixture of 0.8% of P-B in nitrogen is given. The background of the spectra used for computational peak analysis is provided, Figure S6 Surface topography of plasma-treated sample after bending test is given for 1.2% P-B in nitrogen and 7 seconds deposition time.
