# Supplementary material for: Fast Surface Hydrophilization via Atmospheric Pressure Plasma Polymerization for Biological and Technical Applications
Source: Polymers (Basel). 2019 Oct 4;11(10):1613. doi: 10.3390/polym11101613 (PMC6836037; doi:10.3390/polym11101613)
Supplement: Supplementary file 1 [file polymers-11-01613-s001.pdf]

Figure S1 shows the dependency of diiodomethane contact angle (DCA) values on the plasma treatment time. The axes limits are kept the same as in Figure 5 for direct comparison with water contact angle values. Figure S1 presents the DCA at the same conditions as the WCA in Figure 5, i.e., for three different compositions of processing gas, i.e. 0.4, 0.8 and 1.2 % of P-B in nitrogen. The error bars represent the confidence interval of 68 %.

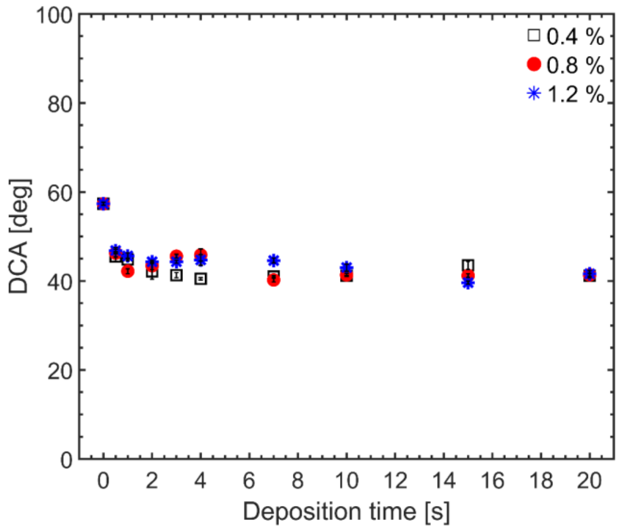

Figure S1 Dependences of diiodomethane contact angle (DCA) values on the plasma treatment time. Values given for three different compositions of processing gas, i.e. 0.4, 0.8 and 1.2 % of P-B in nitrogen are shown.

Figures S2-S4 show the surface free energy (SFE) and corresponding Lifshitz-van der Waals (LW), resp. acid-base (AB) components according to OWRK model implementation in See System SW. For clarity, there are three figures for each processing gas composition, i.e. for 0.4, 0.8 and 1.2 % of P-B in nitrogen, respectively. The error bars of SFE and its components represent the maximal deviation from the mean value computed from the OWRK model. The model considers the mean WCA / DCA values with a confidence interval of 99%.

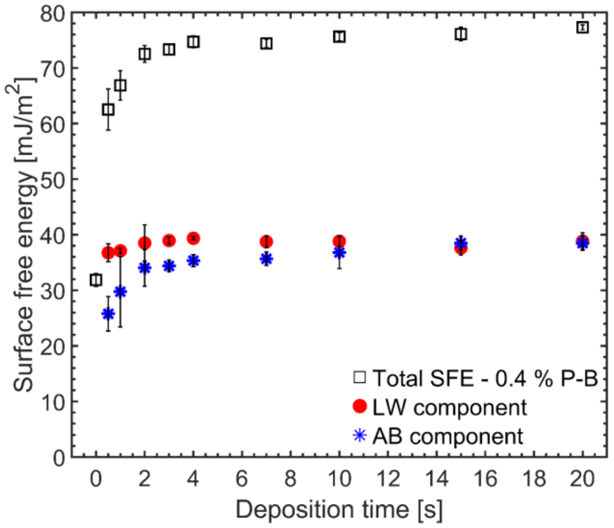

Figure S2 Dependences of surface free energy (SFE) values and corresponding LW, resp. AB components on the plasma treatment time for a gas mixture of 0.4 % of P-B in nitrogen.

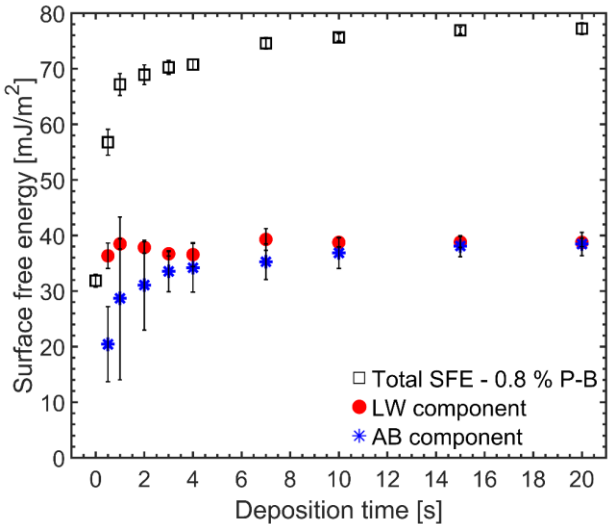

Figure S3 Dependences of surface free energy (SFE) values and corresponding LW, resp. AB components on the plasma treatment time for a gas mixture of 0.8 % of P-B in nitrogen.

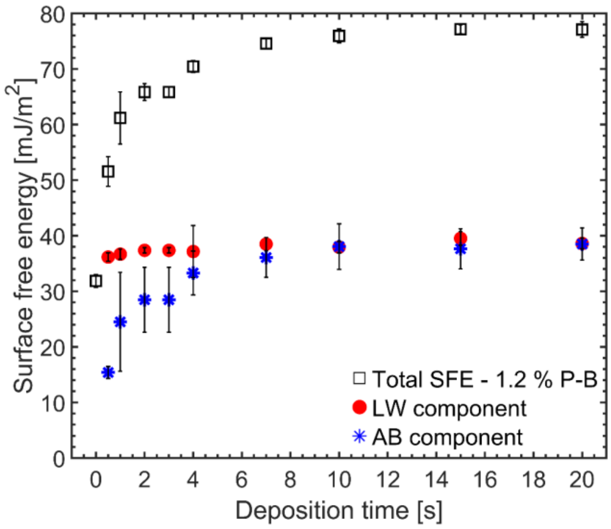

Figure S4 Dependences of surface free energy (SFE) values and corresponding LW, resp. AB components on the plasma treatment time for a gas mixture of 1.2 % of P-B in nitrogen.

Figure S5 shows the XPS survey spectra. There are C1s and O1s peaks identified in the spectrum of the surface in 'as-received' state. The change of the spectrum after thin film deposition is visible. There is a change in the relative intensities of C1s and O1s, and an additional N1s peak is present in the spectrum of the surface covered with a deposited thin film.

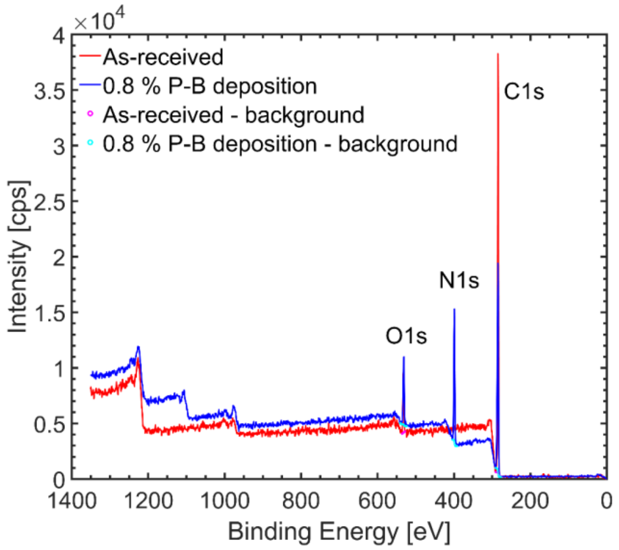

Figure S5 XPS survey spectra of the surface of 'as-received' and after thin film deposition for 10 seconds in a gas mixture of 0.8 % of P-B in nitrogen is given. The background of the spectra used for computational peak analysis is provided.

In Figure 8, the influence of P-B concentration was discussed for the films deposited for 20 seconds, as the film thickness was sufficiently high to produce visible facets. If we compare the films deposited for 7 seconds at 0.4 % P-B concentration with the film deposited at 1.2 % P-B concentration, we can conclude, that the degree of wrinkling and cracking increased with P-B concentration (compare Figure 8(g) and Figure S6). The structure of the film deposited for 7 seconds at 1.2 % P-B concentration is comparable to films deposited at 0.4% P-B and 20 s deposition time conditions, as the thickness of the films becomes comparable (see Figures 8, resp. S6 and deposition rates given in Table 3).

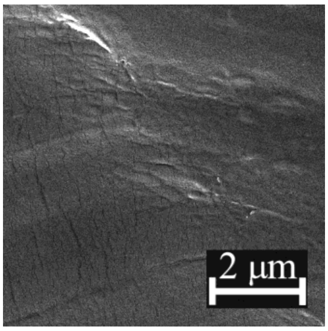

Figure S6 Surface topography of plasma-treated sample after bending test is given for 1.2 % P-B in nitrogen and 7 seconds deposition time.
